# Optimizing SARS-CoV-2 Detection: A Rapid, Cost-Effective Reverse Transcription Polymerase Chain Reaction (RT-PCR) Method Without RNA Extraction

**DOI:** 10.7759/cureus.88654

**Published:** 2025-07-24

**Authors:** Ritu Jain, Ashutosh Rawat, Rahul Ranjan, Tanushree Gahlot, Jagdeepak Gill, Surbhi Gaur, Prachi Sharma, Geetika Nautiyal, Sangeeta Kumari, Manish Teotia

**Affiliations:** 1 Department of Microbiology, Santosh Medical College and Hospital, Santosh Deemed to be University, Ghaziabad, IND

**Keywords:** covid-19, pcr, rt-pcr, sars-cov-2, tata md check rt-pcr xf

## Abstract

Introduction

In December 2019, the emergence of a novel coronavirus, SARS-CoV-2, led to the global COVID-19 pandemic. Early diagnostic efforts included molecular tests such as reverse transcription polymerase chain reaction (RT-PCR). However, testing faced challenges including RNA reagent shortages and prolonged processing times. Antigen card tests, though reliable for positive results, had limitations. Tata MD CHECK RT-PCR XF emerged as a versatile diagnostic assay, capable of detecting SARS-CoV-2 with or without RNA extraction. This facilitated efficient testing amid ongoing resource constraints.

This study was planned to compare the diagnostic accuracy of the Tata MD CHECK RT-PCR XF kit, a direct RT-PCR assay that eliminates the RNA extraction step, with conventional RT-PCR incorporating RNA extraction for detecting SARS-CoV-2 RNA. The direct RT-PCR approach demonstrated a sensitivity of 93.9%, closely aligning with conventional RT-PCR results, and showed comparable performance in lower Ct ranges (0-30), though reduced detection was observed at higher Ct values.

Methods

A cross-sectional study was conducted in the Department of Microbiology, Santosh Medical College, Ghaziabad. Nasopharyngeal and oropharyngeal samples were collected in viral transport media (VTM). Samples were processed in duplicate: one underwent RNA extraction, while the other was directly tested, without RNA extraction, using the Tata MD CHECK RT-PCR XF kit.

Results

A total of 110 samples were evaluated, with 62.7% from males and 37.3% from females, primarily in the 21-40 age group. The Tata MD Direct PCR demonstrated high diagnostic accuracy, with a sensitivity of 93.9%, specificity of 100%, positive predictive value (PPV) of 100%, and negative predictive value (NPV) of 84.4%.

Discussion

This cost-effective and rapid method significantly reduced turnaround times compared to conventional RT-PCR.

## Introduction

Coronaviruses are a group of viruses that typically cause infections in the nose, sinuses, or upper respiratory tract. While most coronaviruses are not dangerous, the WHO identified SARS-CoV-2 as a new type of coronavirus in early 2020, following an outbreak in China in December 2019. This outbreak quickly spread globally, leading to the COVID-19 pandemic [1]. COVID-19 primarily spreads through respiratory droplets and aerosols released when infected individuals cough or sneeze, as well as through prolonged contact with those infected. Symptoms of the disease vary widely, ranging from mild respiratory issues to severe, life-threatening multi-organ failure. The rapid transmission of the virus contributed to its swift global spread [2]. SARS-CoV-2 was initially isolated from a patient's bronchoalveolar lavage (BAL) fluid in China and identified through metagenomic whole-genome sequencing [2]. Within weeks of its discovery, scientists successfully sequenced the complete viral genome, enabling the development of multiple molecular diagnostic assays [2]. Emergency use authorization (EUA) was granted to the CDC's COVID-19 real-time polymerase chain reaction (PCR) test on February 4, 2020 [3].

Reverse transcription PCR (RT-PCR) emerged as the gold standard for diagnosing SARS-CoV-2 infections, but testing efforts were hampered by supply shortages and prolonged processing times. In the early stages of the COVID-19 pandemic, one of the major challenges was the scarcity of RNA extraction reagents. These reagents were costly and hard to procure due to global shortages and lockdowns, further complicating the testing process [4].

Positive results from antigen card tests were generally regarded as reliable and accurate, especially in individuals who were symptomatic or had confirmed exposure. However, negative results were not always definitive, particularly in asymptomatic cases or during the early stages of infection. Because of their lower sensitivity compared to RT-PCR, a negative antigen test often requires confirmation with RT-PCR, which is considered the gold standard due to its higher sensitivity and ability to detect even low viral loads. Consequently, a single negative antigen test result cannot definitively rule out an infection [5]. According to FDA recommendations, to confidently rule out COVID-19, individuals with symptoms should perform two negative antigen tests, while those without symptoms should perform three antigen tests. These tests should be done 48 hours apart [5]. The testing procedure demands significant time and expenses, requiring individuals to purchase numerous antigen test cards.

The Tata MD CHECK RT-PCR XF is a real-time RT-PCR assay designed for rapid and specific detection of SARS-CoV-2 from nasopharyngeal and oropharyngeal specimens. Unlike conventional RT-PCR methods, it supports direct testing without RNA extraction, significantly reducing turnaround time to approximately one hour compared to 2-3 hours for standard protocols [5]. This model stands out for its cost-effectiveness, minimal equipment requirements, and ease of use, making it ideal for basic lab setups and large-scale testing. Its superior speed, affordability, and operational simplicity make it a valuable alternative to traditional RT-PCR assays, especially in resource-limited settings [5].

## Materials and methods

Aim and objectives

Given the urgent need for scalable and efficient diagnostic solutions during infectious disease outbreaks, this study aimed to evaluate a direct RT-PCR assay that omits RNA extraction, a step often constrained by time, cost, and reagent availability. Specifically, the study aimed to detect SARS-CoV-2 RNA using the Tata MD CHECK RT-PCR XF kit and compare its diagnostic accuracy with that of a conventional RT-PCR protocol involving RNA extraction. The objective was to assess whether the direct method could serve as a practical alternative without compromising reliability.

Methods

A cross-sectional study was conducted in the Department of Microbiology at Santosh Medical College, Ghaziabad, over a six-month period from July to December 2024. Using a consecutive sampling strategy, nasopharyngeal and oropharyngeal samples were obtained from symptomatic patients attending inpatient or outpatient services. Eligible participants included individuals of any age who presented with one or more symptoms suggestive of COVID-19, such as fever (≥38°C), cough, shortness of breath, sore throat, anosmia, or ageusia, with symptom onset within the preceding seven days to ensure optimal viral load for RT-PCR analysis. Written informed consent was obtained from adult participants and assent from pediatric patients.

Exclusion criteria included patients who were receiving mechanical ventilation at the time of sampling, had undergone antiviral therapy or COVID-19 vaccination within the previous 48 hours, or whose specimens were inadequately labeled or not transported in viral transport media (VTM) within two hours of collection.

Upon laboratory receipt, each sample was divided and processed in duplicate to allow for method comparison. RNA extraction was carried out using the apsLABS Viral Nucleic Acid Extraction Kit (MAGSPIN-73 PLUS), which employs magnetic bead-based purification. In parallel, direct RT-PCR was performed without an extraction step using the Tata MD CHECK RT-PCR XF diagnostic kit. Conventional RT-PCR was executed according to the GB SARS-CoV-2 RT-PCR kit (General Biologicals Corporation, Taiwan), with Cycle Threshold (Ct) values below 37 considered positive for SARS-CoV-2 RNA. For the direct method, Ct values ≤35 were interpreted as positive per the manufacturer’s guidelines. Results exceeding these thresholds were classified as negative or inconclusive based on internal control performance and repeat testing, where warranted. All RT-PCR reactions were conducted on the BIORAD CFX96 Touch Real-Time PCR system.

Statistical analysis and interpretation

Collected data were compiled using Microsoft Excel, with further statistical analysis performed using SPSS software. Participant data were stratified into predefined age groups to facilitate subgroup analysis. Diagnostic performance metrics, including sensitivity, specificity, positive predictive value (PPV), and negative predictive value (NPV), were calculated to evaluate the efficacy of the Tata MD CHECK RT-PCR XF assay relative to the conventional method. Concordance between Ct values obtained through direct and conventional RT-PCR protocols was assessed. Additionally, Fisher’s exact test was applied to compare detection rates within the higher Ct range (30-40), and 95% confidence intervals were calculated to evaluate proportion differences. A p-value < 0.05 was considered statistically significant.

Ethical clearance was obtained from the Institutional Ethics Committee on 23/09/2023, vide file number F. No. SU/R/2023/2490(11).

## Results

A total of 110 samples were processed in our study. Of the total 110 samples, 62.7% (69/110) were from males, while the remaining 37.3% (41/110) were from females.

Most of the samples tested were within the 21-40 years age group (n = 59), followed by those aged 41-60 years (n = 36), as detailed in Table 1.

**Table 1:** Age distribution of patients whose samples were processed (n = 110).

Age Group (years)	Number of Patients
0-20	6
21-40	59
41-60	36
61-80	8
81-100	1
Total	110

A comparison of Direct PCR (Tata MD) and conventional RT-PCR (n = 110) revealed the following results: 78 samples were identified as true positives, 27 as true negatives, 5 as false negatives, and none as false positives. The performance metrics for Direct PCR (Tata MD) were as follows: sensitivity of 93.9%, specificity of 100%, PPV of 100%, and NPV of 84.4%, as illustrated in Table 2.

**Table 2:** Comparison of Direct PCR (Tata MD) and conventional RT-PCR results (n = 110). RT-PCR: Reverse transcription-polymerase chain reaction.

	Direct PCR (Tata MD) Positive	Direct PCR (Tata MD) Negative	Total
Conventional RT-PCR Positive	78 (True Positive)	5 (False Negative)	83
Conventional RT-PCR Negative	0 (False Positive)	27 (True Negative)	27
Total	78	32	110

Table 3 compares the Ct values obtained through Direct PCR (Tata MD) and conventional RT-PCR, highlighting their respective abilities to detect positive cases across different Ct value ranges. In the lower Ct ranges of 0-20 and 20-30, both methods performed equally well, detecting 35 and 37 positive cases, respectively. However, in the higher Ct range of 30-40, conventional RT-PCR identified 11 positive cases compared to only 6 by Tata MD. This difference suggests reduced sensitivity of the direct method at lower viral loads. To assess this quantitatively, we conducted a comparison of proportions using Fisher’s exact test for the 30-40 Ct range. The difference was found to be statistically significant (p = 0.028), supporting the observation that conventional RT-PCR may be more sensitive in detecting cases with higher Ct values. Additionally, the 95% CI for the proportion difference in this range was 0.03 to 0.58, reinforcing the observed discrepancy.

**Table 3:** Comparison of Ct values from Direct PCR (Tata MD) and conventional RT-PCR. Ct: Cycle Threshold; RT-PCR: Reverse transcription-polymerase chain reaction.

Ct Value Range	Tata MD	Conventional RT-PCR
0-20	35	35
20-30	37	37
30-40	6	11
Total Positive	78	83

## Discussion

SARS-CoV-2 is among the seven types of coronaviruses, which include those responsible for severe illnesses such as Middle East Respiratory Syndrome (MERS) and SARS. The remaining coronaviruses typically cause the common colds that we encounter throughout the year, but they generally do not pose a serious risk to individuals who are otherwise healthy [1]. RT-PCR diagnostic methods are extensively utilized for detecting RNA in infectious agents and are recognized as the most sensitive and specific techniques for their identification [6-7]. To enhance performance and specificity for virological diagnosis, several nucleic acid amplification tests (NAATs) were developed. Among them is real-time PCR, which allows for the detection and quantification of amplified products in each cycle of the PCR reaction [8]. In the nested PCR technique, the product of the initial amplification is used as a template for subsequent amplification, offering high sensitivity and specificity. Conversely, multiplex PCR simultaneously amplifies multiple sequences by utilizing different primer sets in a single reaction [9]. NAAT-based systems are highly effective in detecting disease-causing agents directly from clinical samples. This capability is particularly vital for swiftly identifying microorganisms that are difficult or impossible to culture. These methods have become the gold standard for viral diagnostics, surpassing viral culture due to their wider applicability, greater sensitivity, faster results, and suitability for use in the field [5]. The COVID-19 pandemic put immense pressure on hospitals and commercial laboratories as they struggled to cope with the escalating demand for SARS-CoV-2 testing [10]. One significant challenge to large-scale SARS-CoV-2 testing is the limited availability of extraction kits required for both manual nucleic acid extraction and more comprehensive automated extraction procedures [5]. To tackle this issue, we evaluated the effectiveness of the direct PCR technique (Tata MD CHECK RT-PCR XF kit) by omitting the RNA extraction step and directly utilizing the VTM material for mixing. The Tata MD CHECK RT-PCR XF kit conducts an in vitro real-time RT-PCR qualitative assay. This assay is specifically designed to detect SARS-CoV-2 in human respiratory specimens (nasopharyngeal/oropharyngeal), and it can be used with or without the need for RNA isolation and purification. This kit utilizes a one-step RT-PCR process combined with hydrolysis probe chemistry, leveraging the 5' nuclease activity of Taq DNA polymerase. It amplifies the transcribed DNA through the PCR cycle and detects the resulting PCR product using fluorescence. The assay is designed for the multiplexed detection of the E-gene, N-gene, and RdRP-gene of SARS-CoV-2, along with the human internal control RNase P, in a single reaction. It is compatible with any real-time PCR instrument that has at least four measurement channels (FAM, HEX/VIC, Texas Red/ROX, and CY5). The Limit of Detection (LoD) of Tata MD CHECK RT-PCR XF is 5 copies/μL or 200 copies/mL [5]. The diagnostic performance of the direct RT-PCR approach in our study showed strong agreement with conventional RT-PCR results, achieving a detection rate of 93.9% for SARS-CoV-2-positive cases. This level of sensitivity aligns closely with findings from Santhose NB et al. [5], who reported a comparable detection rate of 92.3% using a similar methodological framework. Notably, the demographic distribution in our study, with a predominance of male participants (62.7%) and a majority within the 21-40 years age group, was also consistent with trends observed in other regional studies, potentially supporting the broader applicability of our results.

The direct PCR method employed in this study is cost-effective, less time-consuming, and easy to perform. This approach significantly reduces the turnaround time (TAT) for results (TAT of direct PCR: approximately 1 hour, compared to 2-3 hours for conventional RT-PCR). It can be executed in a basic laboratory setup with minimal investment. Economically, this method substantially cuts down on machinery costs, consumables, and manpower [11]. Moreover, direct PCR offers safer testing with a lower risk of contamination. Tata MD CHECK is not the only diagnostic platform enabling direct detection of SARS-CoV-2 from clinical samples without manual RNA extraction. Several other technologies, such as the GeneXpert Xpress SARS-CoV-2 assay from Cepheid, utilize cartridge-based systems that automate sample preparation, including RNA extraction, within a closed, hands-free workflow [12]. Cepheid has since introduced the Xpert® Xpress CoV-2/Flu/RSV Plus assay, a multiplex real-time PCR test that simultaneously detects and differentiates SARS-CoV-2, influenza A, influenza B, and respiratory syncytial virus (RSV). This updated formulation replaces the earlier Xpert SARS-CoV-2 and Xpert Flu/RSV assays and is designed to provide rapid results in as little as 25 minutes, with built-in redundancy to mitigate the impact of viral mutations [13].

Limitations of Tata MD CHECK RT-PCR XF kit and future directions

This study acknowledges several limitations inherent to the evaluation of the Tata MD CHECK RT-PCR XF kit. First, the assay’s performance was validated exclusively on nasopharyngeal and oropharyngeal swab samples; it remains untested on other respiratory specimen types such as sputum or bronchoalveolar lavage, which may hold clinical relevance in certain settings. Future validation studies should explore the kit's applicability across diverse specimen types to enhance diagnostic versatility. Second, the impact of pharmacological interventions, including vaccines, antivirals, antibiotics, chemotherapeutics, and immunomodulatory agents, was not assessed. These factors may influence viral load dynamics or assay performance and warrant systematic evaluation in future cohorts. Third, while the kit demonstrated high concordance with conventional RT-PCR, the absence of an RNA concentration step introduces sensitivity limitations, particularly in low viral load samples. The reduced input of template RNA may lead to delayed Ct values or undetected positives [5]. To mitigate this, future research could investigate pre-analytical sample concentration strategies or enhancements in assay chemistry to bolster template recovery in direct amplification workflows. Furthermore, Ct value interpretation may be challenging in resource-limited settings due to variability in laboratory infrastructure, lack of standardized calibration, and limited access to internal controls, factors that could compromise the clinical reliability of borderline results. Addressing these operational constraints is essential to ensure consistent and meaningful diagnostic output in low-resource environments. Lastly, our study did not correlate Ct values with clinical parameters such as symptom severity, disease progression, or treatment history. This restricts the interpretability of viral load measurements in clinical decision-making. Prospective studies integrating patient-level metadata and longitudinal follow-up are recommended to clarify the prognostic and diagnostic significance of Ct values in real-world applications.

In summary, the Tata MD CHECK RT-PCR XF direct assay offers a practical alternative to conventional extraction-based RT-PCR for SARS-CoV-2 detection. Despite a slight reduction in sensitivity, its high diagnostic concordance (93.9%), faster turnaround, cost-efficiency, and operational simplicity make it especially advantageous in resource-limited settings [14]. The direct RT-PCR method, by bypassing RNA extraction, substantially shortens TAT and reduces dependency on extraction reagents, particularly beneficial during pandemic peaks or supply constraints. While reduced sensitivity due to absent sample concentration remains a limitation, the method’s operational efficiency highlights its utility in scaling diagnostic capacity amid public health emergencies [15].

## Conclusions

This study demonstrates that the direct RT-PCR approach using the Tata MD CHECK RT-PCR XF kit is a viable alternative to conventional RNA extraction-based testing methods for SARS-CoV-2 detection. Although marginally less sensitive, the method achieved a high diagnostic concordance of 93.9% with traditional RT-PCR assays. By streamlining workflow and eliminating the extraction step, the protocol reduced turnaround time and operational complexity. Moreover, it offered appreciable cost advantages, reinforcing its relevance in resource-limited settings where affordability, efficiency, and accessibility are essential.

The direct RT-PCR technique also reduces dependency on specialized extraction reagents, an operational advantage during pandemic surges and supply disruptions. While certain limitations persist, such as reduced sensitivity stemming from the absence of sample concentration, the method’s overall simplicity, speed, and cost-effectiveness underscore its potential to enhance diagnostic reach during public health emergencies.

## References

[1] World Health Organization. Coronavirus disease (COVID-19). https://www.who.int/health-topics/coronavirus.

[2] Uhteg K, Jarrett J, Richards M (2020). Comparing the analytical performance of three SARS-CoV-2 molecular diagnostic assays. J Clin Virol.

[3] Lu X, Wang L, Sakthivel SK (2020). US CDC real-time reverse transcription PCR panel for detection of severe acute respiratory syndrome coronavirus 2. Emerg Infect Dis.

[4] Cameron A, Pecora ND, Pettengill MA (2021). Extraction-free methods for the detection of SARS-CoV-2 by reverse transcription-PCR: a comparison with the Cepheid Xpert Xpress SARS-CoV-2 assay across two medical centers. J Clin Microbiol.

[5] Santhose NB, Murali KS, Avinash D (2024). Faster and cost-effective extraction free method for SARS-CoV-2 detection by RT-PCR. Int J Adv Res.

[6] Tang YW, Procop GW, Persing DH (1997). Molecular diagnostics of infectious diseases. Clin Chem.

[7] Liu S, Chai X, Liu C (2024). Sensitivity analysis of RT-qPCR and RT-ddPCR for SARS-CoV-2 detection with mutations on N1 and E primer-probe region. Microbiol Spectr.

[8] Costa J (2004). Reaccion en cadena de la polimerasa (PCR) a tiempo real. Enfermedades Infecciosas y Microbiología Clínica.

[9] Erlich HA (1989). Polymerase chain reaction. J Clin Immunol.

[10] Bruce EA, Huang ML, Perchetti GA (2020). Direct RT-qPCR detection of SARS-CoV-2 RNA from patient nasopharyngeal swabs without an RNA extraction step. PLoS Biol.

[11] Wang M, Chu Y, Qiang L (2022). Rapid, amplification-free and high-throughput SARS-CoV-2 RNA detection via a reduced-graphene-oxide based fluorescence assay. Sens Diagn.

[12] Bel Hadj Ali I, Souguir H, Melliti M (2024). Rapid detection of SARS-CoV-2 RNA using a one-step fast multiplex RT-PCR coupled to lateral flow immunoassay. BMC Infect Dis.

[13] Johnson G, Zubrzycki A, Henry M (2021). Clinical evaluation of the GeneXpert® Xpert® Xpress SARS-CoV-2/Flu/RSV combination test. J Clin Virol Plus.

[14] Pearson JD, Trcka D, Lu S (2021). Comparison of SARS-CoV-2 indirect and direct RT-qPCR detection methods. Virol J.

[15] Song Y, Huang P, Yu M (2023). Rapid and visual detection of SARS-CoV-2 RNA based on reverse transcription-recombinase polymerase amplification with closed vertical flow visualization strip assay. Microbiol Spectr.

